# Corneal irregularity of the anterior and posterior surface in patients with limbal stem cell deficiency evaluated with anterior-segment optical coherence tomography

**DOI:** 10.1007/s10384-025-01242-y

**Published:** 2025-07-21

**Authors:** Hiroki Goto, Takashi Ono, Yukako Taketani, Yuito Abe, Mikiko Kimakura, Tetsuya Toyono, Makoto Aihara, Takashi Miyai

**Affiliations:** Department of Ophthalmology, University of Tokyo Hospital, 7-3-1 Hongo, Bunkyo-ku, Tokyo, 113-8655 Japan

**Keywords:** Astigmatism, Corneal imaging, Corneal tomography, Ocular surface, Stem cell deficiency

## Abstract

**Purpose:**

To evaluate corneal anterior and posterior irregularities due to limbal stem cell deficiency (LSCD) based on staging using Fourier harmonic analysis with anterior segment optical coherence tomography (AS-OCT).

**Study design:**

Retrospective observational study.

**Methods:**

Patients with LSCD and those without anterior segment disease (controls), examined using AS-OCT, were retrospectively included. Based on Fourier harmonic analysis of the central 3 mm, spherical components, regular astigmatism, asymmetry components, and higher-order irregularities of the anterior, posterior, and total corneas were compared between the groups.

**Results:**

We analyzed 72 eyes of 72 patients—25 eyes of 25 patients (63.0 ± 15.8 years) with LSCD and 47 eyes of 47 patients (66.8 ± 9.5 years) in the control group. Regular astigmatism, asymmetry components, and higher-order irregularities of the anterior, posterior, and total corneas were higher in LSCD than in the control group (all P < 0.001). Based on LSCD staging, asymmetry components of the total cornea were higher in LSCD stages I, II, and III than in the controls (P = 0.034, P < 0.001, and P < 0.001, respectively). Additionally, higher-order irregularity was larger in stages II and III of LSCD than in the controls (all P < 0.001). The higher-order irregularity in LSCD significantly correlated with best-corrected visual acuity (P = 0.034).

**Conclusion:**

Corneal irregularities in the anterior and posterior corneas increased in patients with LSCD and contributed to best-corrected visual acuity. Even in patients at stage I, where the lesion spared the central 5 mm, an increase in asymmetric astigmatism within the central 3 mm was observed.

## Introduction

Limbal stem cell deficiency (LSCD) is a condition in which the corneal epithelial stem cells are depleted, and their function is impaired because of damage to the corneal limbus [1–3]. LSCD causes conjunctival invasion of the cornea, induces corneal neovascularization, and is associated with pain, photophobia, and decreased vision [3]. Exogenous, inflammatory, and congenital mechanisms cause LSCD. Exogenous mechanisms include ocular surface burns, which are the leading cause of LSCD [4], and long-term contact lens use [5]. The inflammatory mechanism involves Stevens–Johnson syndrome (SJS)/toxic epidermal necrolysis (TEN) and ocular cicatricial pemphigoid (OCP) [4, 6]. Congenital aniridia is a representative congenital mechanism [7].

In patients with LSCD, the cornea is covered with an invasive conjunctiva; however, the visual axis is impaired in the progressed situation. Furthermore, even in the early stages, corneal topographic changes due to asymmetric corneal opacification or vascular invasion have been observed in the clinic. Anterior-segment optical coherence tomography (AS-OCT) is a non-invasive examination that allows measurement of corneal thickness and astigmatism *in vivo* and can provide images of the corneal limbus and help diagnose LSCD [8, 9]. Fourier harmonic analysis of the corneal surface is a valuable method for analyzing corneal shape data through decomposition into four factors (spherical component, regular astigmatism, asymmetry component, and higher-order irregularity). It has been broadly used for detailed assessments of corneal topography in anterior eye diseases such as post-corneal transplantation [10], pterygium [11], and bullous keratopathy [12].

Currently, only a few reports have evaluated astigmatism in detail in patients with LSCD [13]. No clinical study has evaluated corneal irregularities following an objective examination based on Fourier harmonic analysis using AS-OCT, nor has there been any report evaluating anterior and posterior irregular astigmatism by LSCD staging. We aimed to clarify whether corneal irregularities progress in parallel with LSCD progression and whether corneal irregularity in LSCD contributes to patients' visual acuity.

## Subjects and methods

### Patients

This retrospective observational study was approved by the Institutional Review Board of the Research Ethics Committee of the University of Tokyo School of Medicine (Examination No. 2217) and adhered to the tenets of the Declaration of Helsinki. Informed consent was obtained from all the participants through the opt-out method.

Outpatients with LSCD who visited the University of Tokyo Hospital between April 2022 and October 2023 and whose corneas were examined using AS-OCT (CASIA2; Tomey Corporation) were included in this study (LSCD group). Patients with insufficient medical information were excluded. Poor AS-OCT images were removed based on the image-quality system application provided in CASIA2. Medical charts were retrospectively reviewed to obtain patient background, best-corrected visual acuity (BCVA), and corneal data. If both eyes were affected, the eye with the poorer BCVA was selected. Control eyes of patients who visited the same hospital during the same period with no history of corneal or conjunctival diseases, averaged corneal astigmatism within − 1.5 D, central corneal thickness of 500–560 μm, no history of rigid gas-permeable (RGP) lens wear within one month, and BCVA ≤ 0 (logMAR) were also included (control group). LSCD was diagnosed based on global consensus [3]. The severity of LSCD was graded as stages I, II, or III, as previously reported: stage I, normal corneal epithelium within the central 5-mm zone of the cornea; stage II, the central 5-mm zone of the cornea is affected; and stage III, the entire corneal surface is affected [3].

### Examinations

Corneal irregularity was evaluated using AS-OCT data. Fourier harmonic analysis of the corneal topographic data was conducted as previously reported [14]. The reconstructed axial refractive power data, based on corneal tomography, were decomposed using a series of trigonometric components. The dioptric powers on mire ring i, F_i_(σ), were transformed into the trigonometric components of the form with the Fourier series harmonic analysis program included in the equipment, as follows:

$${\text{F}}_{\text{i}}\left(\upsigma \right)={a}_{0}+{c}_{1}\text{cos}\left(\sigma -{\alpha }_{1}\right)+{c}_{2}\text{cos}2\left(\sigma -{\alpha }_{2}\right)+ {c}_{3}\text{cos}3\left(\sigma -{\alpha }_{3}\right)+\dots +{c}_{n}\text{cos}n\left(\sigma -{\alpha }_{n}\right)$$

In this equation, a_0_ is the spherical component of the ring, 2 × c_1_ is the asymmetry component (i.e., tilt or decentration), 2 × c_2_ is regular astigmatism, and the summation of c_3_–c_n_ refers to higher-order irregularity components. These four parameters were examined in the cornea within a 3-mm diameter using a built-in program in AS-OCT, and the values were averaged. The four components for the anterior and posterior cornea and the total value were assessed for each LSCD stage. We conducted multiple examinations on each case with adequate fixation. We minimized the influence of the adherent eyelid as much as possible and extracted only the data with high reproducibility from the multiple examinations.

### Statistical analysis

The Mann–Whitney U test was used to compare the values between the two groups. The Kruskal–Wallis test with Dunn's multiple comparisons was used to compare the values among the LSCD stages. Linear regression analysis was used to examine the correlation between the four parameters of the Fourier harmonic analysis and BCVA. The statistical analyses were performed using GraphPad Prism 9.5.1 (GraphPad Software). Statistical significance was set at P < 0.05. All values are described as the mean ± standard deviation.

## Results

In total, 72 eyes of 72 patients were included in this study. The LSCD group comprised 25 eyes of 25 patients (16 men and nine women; age, 63.0 ± 15.8 years). The control group comprised 47 eyes of 47 patients (26 men and 21 women; age, 66.8 ± 9.5 years). The demographic characteristics of the two groups are presented in Table 1. The causative diseases of LSCD were SJS/TEN in six patients, chemical trauma in six patients, aniridia/chromosomal abnormality in four patients, facial nerve paralysis/lagophthalmos in two patients, OCP in one patient, and unknown causes in six patients. The disease stages of the patients were: stage I, five patients; stage II, nine patients; and stage III, 11 patients.

**Table 1.** Demographic background of limbal stem cell deficiency (LSCD) and control groups

	LSCD group	Control group	*P*-value
Eyes (n)	25	47	–
Age (years)	63.0 ±15.8 (19−88)	66.8 ± 9.5 (48–87)	0.206
Male/female (eyes)	16/9	26/21	0.477
Central corneal thickness (μm)	537.6 ± 140.6 (266–820)	530.5 ± 14.7 (506–559)	0.821
Best-corrected visual acuity (logMAR)	1.65 ± 0.96	− 0.063 ± 0.041	< 0.001

The average corneal astigmatism of the anterior cornea was significantly higher in the LSCD group than in the control group (7.112 ± 6.07 D and 0.896 ± 0.649 D, respectively; P < 0.001). The average corneal astigmatism of the posterior cornea was also significantly higher in the LSCD group than in the control group (0.608 ± 0.492 D and 0.338 ± 0.149 D, respectively; P < 0.001). In the Fourier harmonic analysis of the anterior corneal surface, the indices for regular astigmatism, asymmetry component, and higher-order irregularity were higher in the LSCD group than in the control group (all P < 0.001; Table 2). Similarly, the analysis of the posterior corneal surface yielded higher values for regular astigmatism, asymmetry component, and higher-order irregularity in the LSCD group than in the control group (all P < 0.001; Table 2). Accordingly, in the total corneal analysis, the values of these three parameters were higher in the LSCD group than in the control group (all P < 0.001; Table 2).

**Table 2.** Comparison in corneal irregularity of the anterior, posterior, and total cornea between the limbal stem cell deficiency (LSCD) and control groups

		LSCD group	Control group	*P*-value
Anterior cornea	Spherical components (D)	54.21 ± 6.52	49.00 ± 0.58	0.060
	Regular astigmatism (D)	4.60 ± 2.33	0.50 ± 0.10	< 0.001
	Asymmetry components (D)	5.50 ± 2.08	0.26 ± 0.06	< 0.001
	Higher-order irregularity (D)	2.09 ± 1.30	0.16 ± 0.02	< 0.001
Posterior cornea	Spherical components (D)	−6.19 ± 0.61	−6.21 ± 0.08	0.887
	Regular astigmatism (D)	0.39 ± 0.23	0.17 ± 0.02	< 0.001
	Asymmetry components (D)	0.38 ± 0.25	0.05 ± 0.01	< 0.001
	Higher-order irregularity (D)	0.21 ± 0.16	0.02 ± 0.002	< 0.001
Total cornea	Spherical components (D)	48.15 ± 6.80	42.9 ± 0.52	0.087
	Regular astigmatism (D)	4.64 ± 2.52	0.40 ± 0.08	< 0.001
	Asymmetry components (D)	4.94 ± 1.92	0.26 ± 0.06	< 0.001
	Higher-order irregularity (D)	2.15 ± 1.37	0.15 ± 0.02	< 0.001

**Fig. 1:**
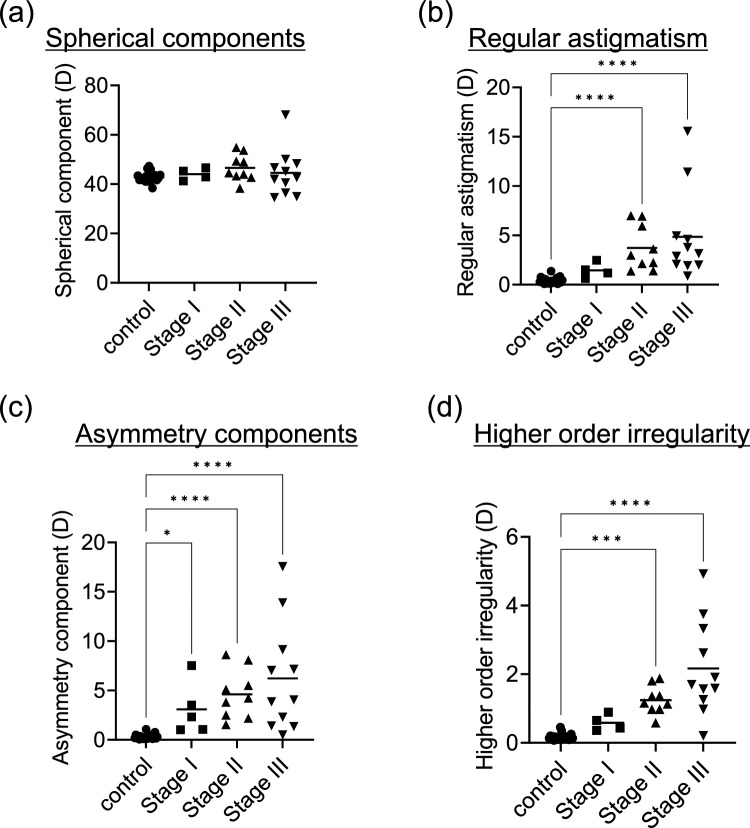
Comparison between the four components of the Fourier harmonic analysis and LSCD stages. **a** Spherical components and LSCD stages. No significant differences were observed between the control group and LSCD stages I, II, and III. **b** Regular astigmatism and LSCD stages. Regular astigmatism was higher in the stage II and III groups than in the control group (all *P* < 0.001). **c** Asymmetry components and LSCD stages. Asymmetry components were greater in stages I, II, and III than in the control group (*P* = 0.034, *P* < 0.001, and* P* < 0.001, respectively). **d** Higher-order irregularity and LSCD stages. Higher-order irregularity was greater in stages II and III than in the control group (all *P* < 0.001). LSCD: limbal stem cell deficiency.

Next, we evaluated variations in the four parameters of the Fourier harmonic analysis based on the LSCD stages. There was no difference between the control group and stages I–III of LSCD regarding the spherical component (Fig. 1a). In contrast, a significant increase in regular astigmatism was noted in stage II and stage III LSCD compared to the control group (P < 0.001; Fig. 1b). The asymmetry component was higher in LSCD stages I, II, and III than in the control group (P = 0.034, < 0.001, and < 0.001, respectively; Fig. 1c), suggesting that the values increased step-by-step as the stage progressed, although there was no significant difference between the other groups. Higher-order irregularity was larger in LSCD stages II and III than in the control group (all P < 0.001; Fig. 1d).

**Fig. 2:**
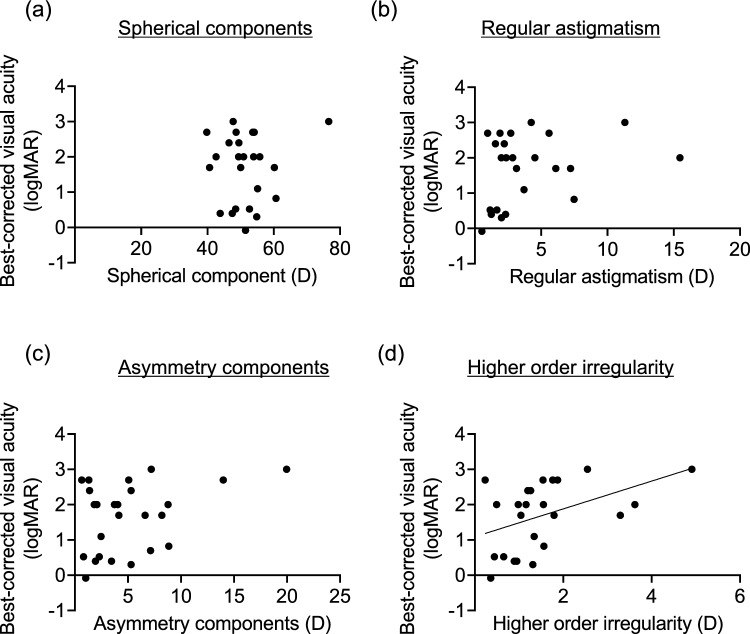
Relationship between the four components of the Fourier harmonic analysis in the anterior cornea and best-corrected visual acuity of patients. **a** Spherical components of the anterior cornea and best-corrected visual acuity. No significant relationship was observed. **b** Regular astigmatism of the anterior cornea and best-corrected visual acuity. No significant relationship was observed. **c** Asymmetry component of the anterior cornea and best-corrected visual acuity. No significant relationship was observed. **d** Higher-order irregularity of the anterior cornea and best-corrected visual acuity. Best-corrected visual acuity significantly correlated with higher-order irregularity in patients with LSCD (R^2^ = 0.204, *P* = 0.027)

**Fig. 3:**
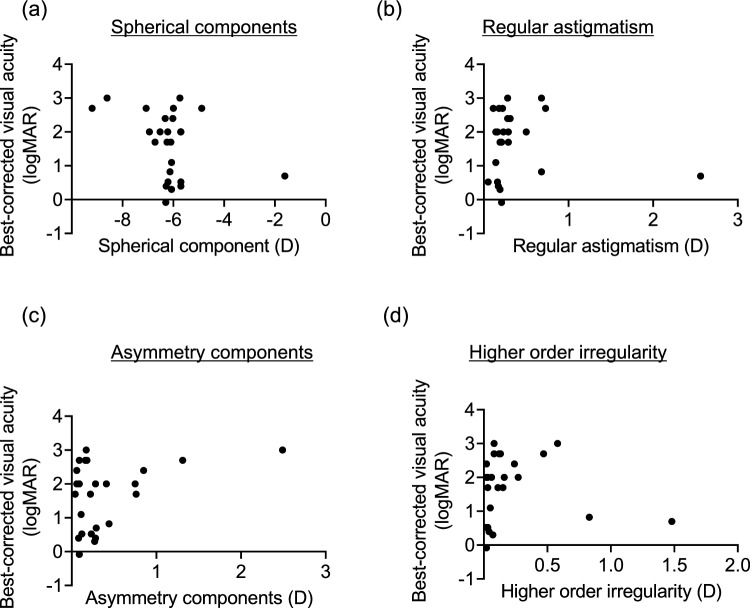
Relationship between the four components of the Fourier harmonic analysis in the posterior cornea and best-corrected visual acuity of patients. **a** Spherical components of the posterior cornea and best-corrected visual acuity. No significant relationship was observed. **b** Regular astigmatism of the posterior cornea and best-corrected visual acuity. No significant relationship was observed. **c** Asymmetry component of the posterior cornea and best-corrected visual acuity. No significant relationship was observed. **d** Higher-order irregularity of the posterior cornea and best-corrected visual acuity. No significant relationship was observed.

**Fig. 4:**
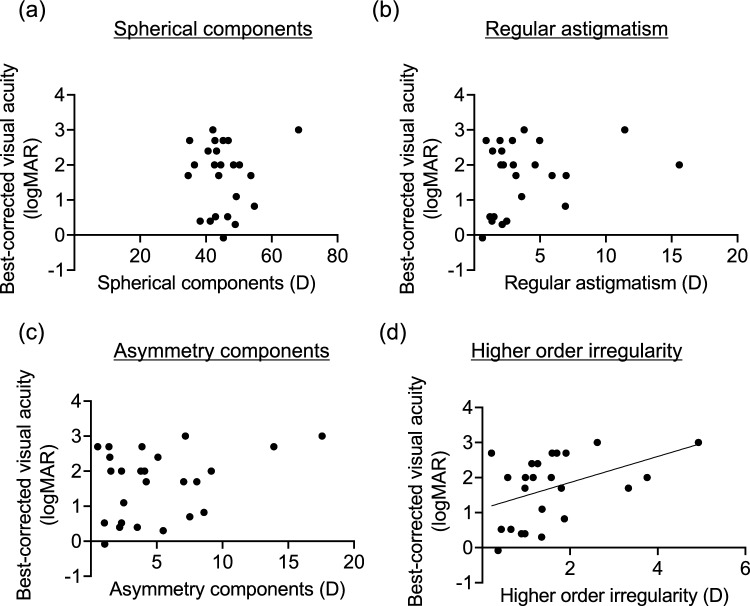
Relationship between the four components of the Fourier harmonic analysis in the total cornea and best-corrected visual acuity of patients. **a** Spherical components of the total cornea and best-corrected visual acuity. No significant relationship was observed. **b** Regular astigmatism of the total cornea and best-corrected visual acuity. No significant relationship was observed. **c** Asymmetry component of the total cornea and best-corrected visual acuity. No significant relationship was observed. **d** Higher-order irregularity of the total cornea and best-corrected visual acuity. Best-corrected visual acuity significantly correlated with higher-order irregularity in patients with LSCD (R^2^ = 0.190,* P* = 0.034).

**Fig. 5:**
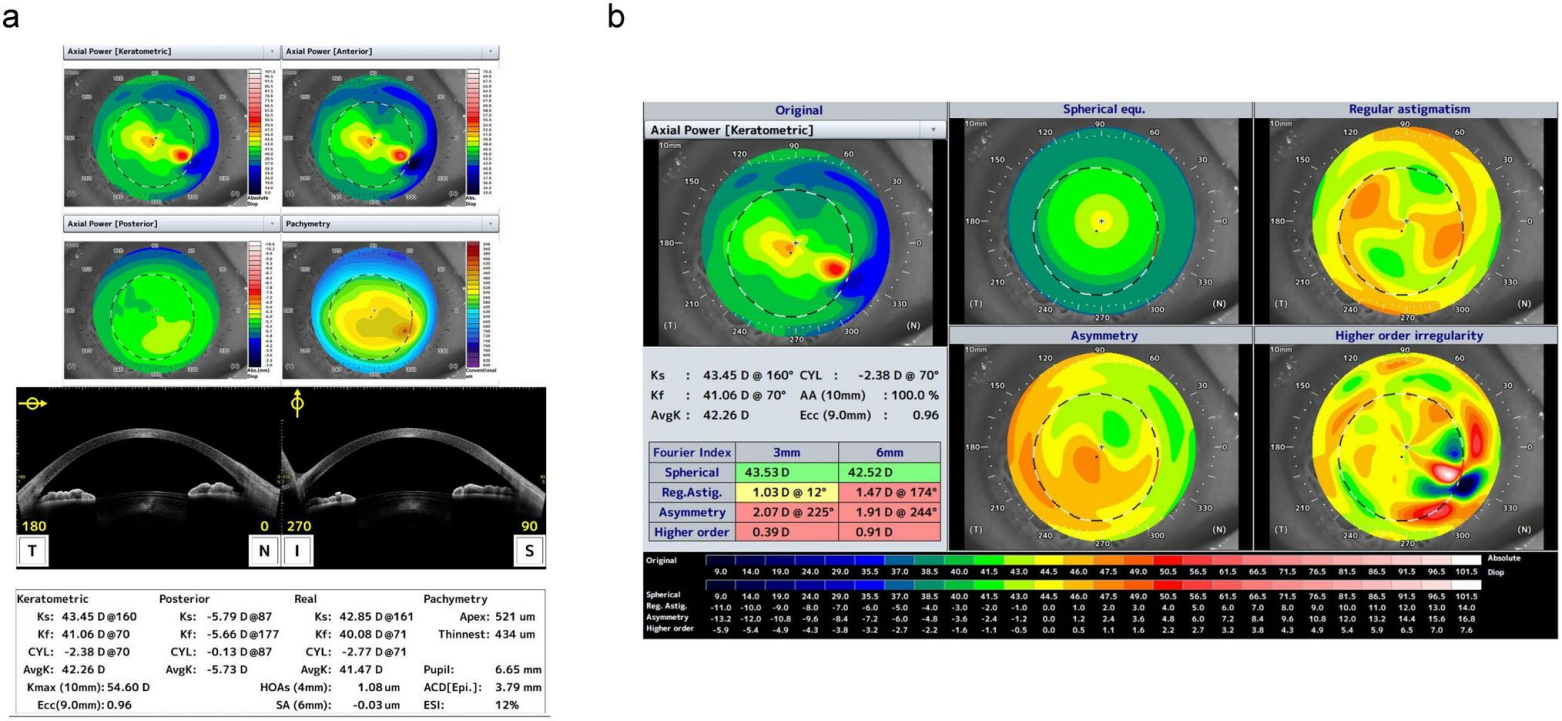
Anterior segment optical coherence tomography images of a representative LSCD stage I case. **a** An LSCD stage I case demonstrates corneal irregularity on the anterior surface. **b** Fourier harmonic analysis detected asymmetry and higher-order irregularity was elevated on the anterior surface.

**Fig. 6:**
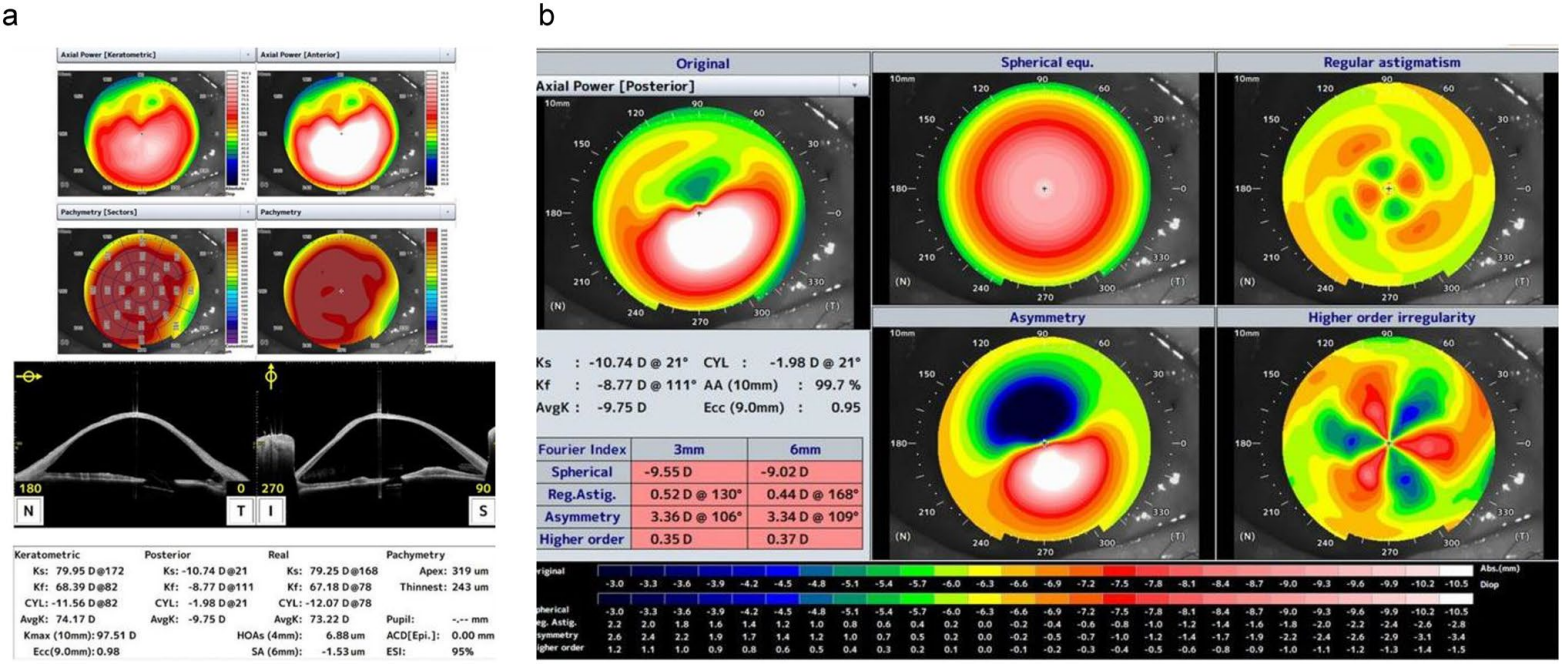
Anterior segment optical coherence tomography images of a representative LSCD stage III case. **a** Tomography and representative indices of the LSCD case reveal anterior irregular astigmatism and corneal thinning similar to keratoconus. **b** The Fourier maps of the case demonstrate posterior increased regular astigmatism, asymmetry, and higher-order irregularity.

In the LSCD cases, the spherical component measurements from the anterior cornea were not associated with BCVA (Fig. 2a). Regular astigmatism and the asymmetric component also did not correlate with BCVA (Fig. 2b and c). Conversely, higher-order irregularity was significantly related to BCVA (P = 0.027), suggesting that corneal irregularity contributes to visual acuity impairment in patients with LSCD. No significant relationship was observed between the parameters of the posterior corneal surface and BCVA (Fig. 3a–d). Lastly, no significant associations were noted between BCVA and the spherical component, regular astigmatism, and asymmetry component for the total cornea (Fig. 4a–c), whereas higher-order irregularity correlated significantly with BCVA (P = 0.034, Fig. 4d). A representative topographic image from AS-OCT of a patient with LSCD stage I is shown in Fig. 5a, while the image for stage III is shown in Fig. 6a; the results of the Fourier harmonic analysis for the same patient are presented in Figs. 5b and 6b, respectively. Additionally, we compared the results based on the presence or absence of corneal stromal opacity. There were no differences in any component of the Fourier analysis when comparing patients with corneal stromal opacity to those without it (Table 3).

**Table 3.** Comparison of patients with and without corneal stromal opacity in the limbal stem cell deficiency (LSCD) groups

		Corneal stromal opacity (–)	Corneal stromal opacity (+)	*P*-value
	N (eyes)	6	19	–
	Best-corrected visual acuity (logMAR)	1.14 ± 0.89	1.82 ± 0.95	0.145
Anterior cornea	Spherical components (D)	62.56 ± 23.92	51.01 ± 7.92	0.294
	Regular astigmatism (D)	7.53 ± 9.04	3.9 ± 3.73	0.379
	Asymmetry components (D)	5.21 ± 3.25	5.07 ± 4.89	0.938
	Higher-order irregularity (D)	3.31 ± 5.45	1.65 ± 1.20	0.490
Posterior cornea	Spherical components (D)	−5.41 ± 1.89	−6.4 ± 1.01	0.264
	Regular astigmatism (D)	0.64 ± 0.97	0.28 ± 0.17	0.398
	Asymmetry components (D)	0.21 ± 0.14	0.45 ± 0.60	0.114
	Higher-order irregularity (D)	0.43 ± 0.60	0.13 ± 0.16	0.275
Total cornea	Spherical components (D)	57.27 ± 25.66	44.74 ± 7.36	0.288
	Regular astigmatism (D)	7.83 ± 9.95	3.86 ± 3.74	0.378
	Asymmetry components (D)	5.23 ± 3.19	4.94 ± 4.51	0.865
	Higher-order irregularity (D)	3.49 ± 5.76	1.66 ± 1.22	0.474

## Discussion

Fourier harmonic analysis of the corneal surface demonstrated that regular and irregular corneal astigmatism was significantly higher in the LSCD group than in the control group. An increase in irregular astigmatism, which is challenging to correct with spectacles, was observed in the early stages of LSCD, and higher-order irregularity increased significantly in stages II and III, suggesting that irregular corneal astigmatism tended to worsen as LSCD progressed. This may have been caused by irregular epithelial thickness, stromal thinning, peripheral neovascularization of the cornea, corneal epithelial defects, and corneal scarring. In the corneas of patients with LSCD, the conjunctiva gradually invades from the periphery to the central cornea. The conjunctiva subsequently invades the pupil in stages II and III of LSCD, blocking the passage of light and leading to vision impairment. In contrast, in stage I of LSCD, the invading conjunctiva does not cover the transparent central cornea but changes the three-dimensional structure of the corneal shape with pulling power. The same phenomenon has been observed in cases of pterygium, where the conjunctiva extends into the cornea, similar to the events in LSCD, resulting in an increase in corneal irregularity with disease progression [15]. Conjunctivae that extend close to the pupil are reported to alter the shape of the cornea even if they are not directly attached [16]. Furthermore, LSCD affected the anterior corneal surface, and topographic changes were observed on the posterior surface, as presented in Table 2. The posterior surface of the cornea, the endothelial cell layer, is not directly invaded via neovascularization owing to the loss of function of limbal stem cells. Fig. 5a and b illustrate a representative case of LSCD with evidence from both the anterior and posterior surfaces. The tomography results in Fig. 5a show corneal thinning and protrusion similar to keratoconus, while the posterior Fourier index map in Fig. 5b shows anterior protrusion of the posterior corneal surface and increased posterior regular astigmatism, asymmetry, and higher-order irregularity. Based on these observations, we suggest that the progression of the conjunctiva onto the cornea on the anterior surface distorts the entire cornea and that its effects extend to the posterior surface of the cornea.

Our clinical study demonstrates that greater irregular corneal astigmatism, especially higher-order irregularity, explains the low visual acuity in patients with LSCD. It is challenging to evaluate corneal astigmatism because the mire ring in keratometry is distorted in patients with rough ocular surfaces. Here, the newly developed AS-OCT enabled us to evaluate the corneal topography in the central 3-mm diameter of the anterior and posterior cornea. Previously, Ibrahim et al. reported that high-order aberrations correlated highly with visual acuity in patients with SJS and TEN [17]. However, there is no detailed information regarding corneal anterior and posterior astigmatism in patients with LSCD. Moreover, several patients with LSCD also have corneal opacity due to primary diseases (SJS/TEN, OCP, or chemical burns), because higher-order aberrations are sometimes affected by corneal opacity. Therefore, we focused on assessing corneal refractive characteristics using Fourier harmonic analysis. Fourier analysis provides a more detailed assessment of corneal shape, including spherical components, regular astigmatism, asymmetry components, and higher-order irregularity. Both Fourier analysis and Zernike polynomials are valid methods for analyzing corneal shape, and both corneal analysis techniques are clinically useful. In the current research, we chose Fourier analysis for the following reasons: (1) Fourier analysis enables us to decompose the corneal shape comprehensively, breaking down the corneal surface into four parameters in a form that clinical ophthalmologists can easily understand; (2) Fourier analysis is correlated with visual outcomes: spherical components and regular astigmatism can be corrected with spectacles, whereas asymmetry components and higher-order irregularity cannot and require correction using contact lenses. This distinction is important for patients, as understanding whether they need glasses or contact lenses is clinically valuable. Based on the results of Fourier analysis, ophthalmologists can select and prescribe the appropriate visual aids; Furthermore, Fourier analysis allows for the classification of the causes of irregular astigmatism, distinguishing whether they result from asymmetry or higher-order irregularity, making the interpretation more intuitive. (3) Fourier components can be displayed as color-coded maps, facilitating easier interpretation of corneal shape characteristics for both doctors and patients. At the same time, it is also important to understand aberrations by Zernike polynomials, as they allow for a detailed classification of optical distortions, including coma and trefoil, and provide a standardized approach to wavefront analysis. Further analysis for patients with LSCD is needed in the future to better characterize the optical impact of corneal irregularities.

In this study, only higher-order irregularity was correlated with visual acuity. However, it seemed likely that asymmetry components would be associated with visual acuity because these components are not corrected with spectacles. In the present study, Fig. 2c shows a tendency for visual acuity to worsen as asymmetry components in the anterior cornea increased, although this was not significant (P = 0.083). In Fig. 3c, P = 0.067 was not significant either, but there was a tendency for visual acuity to worsen as the asymmetry components of the posterior corneal surface increased. Since the number of patients in this retrospective observational study was only 25, and the ocular surface shape of LSCD patients is highly variable, further studies with a larger number of patients are needed.

Treatment for irregular astigmatism not corrected by spectacles is limited. Although RGP lenses generally provide better outcomes for patients with high corneal astigmatism, they cause mechanical trauma, dry eye, hypoxia, and preservative toxicity that lead to stress in the corneal epithelial cells and can induce LSCD; therefore, RGP lenses should be avoided [5, 18, 19]. Alternatively, scleral lenses have been developed to retain fluid on the irregular surface of the cornea and maintain visual acuity [20, 21]. Correction of irregular corneal astigmatism improves visual acuity in the scleral lens, but the scleral lens causes hypoxia on the ocular surface, possibly leading to the progression of LSCD [21]. Therefore, these lenses should be used cautiously, with the degree of progression being observed frequently. Cultivated limbal epithelial cell sheet transplantation, another treatment option in regenerative medicine, was developed to reconstruct the ocular surface [22]. Despite these advances, it would be desirable if LSCD progression could be stopped before reaching a stage where the irregular astigmatism becomes more marked. Furthermore, once treatment for advanced cases is established, there is potential for improvement in corneal irregularity, leading to better visual acuity.

The current study had some limitations. First, cataracts and macular hypoplasia were often noted in patients with aniridia, and there was a significant difference in BCVA between the LSCD and control groups. However, sex, age, and central corneal thickness showed no intragroup differences. Therefore, we only analyzed the relationship between the four components of the Fourier harmonic analysis and BCVA in the LSCD group. Second, patients with SJS/TEN or OCP sometimes have symblepharon, which attaches to the conjunctiva near the cornea and the eyelids and can affect corneal topography. To account for this issue, we carefully examined the patients' eyes and applied as little extra pressure as possible to the eyeball after opening the eyelids. Third, in the present study, we were unable to determine which corneal factors—epithelium or stroma—have the greatest influence on corneal irregular astigmatism, as we could not accurately assess epithelial and stromal thickness using anterior segment optical coherence tomography. Fourth, visual function is influenced by both astigmatism related to corneal shape and reduced corneal transparency. Although we could not evaluate the degree of corneal opacity in this study, it is possible that it affected visual acuity. Since AS-OCT can evaluate densitometry, future studies should consider densitometry values according to LSCD staging.

In conclusion, an increase in irregular astigmatism was observed on both the anterior and posterior corneal surfaces in patients with LSCD, regardless of the stage, using Fourier harmonic analysis of AS-OCT.

## Data Availability

The data supporting the findings of this study are available from the corresponding author upon reasonable request.

## References

[1] Davanger M, Evensen A. Role of the pericorneal papillary structure in renewal of corneal epithelium. Nature. 1971;229:560–1. 4925352 10.1038/229560a0

[2] Deng SX, Kruse F, Gomes JAP, Chan CC, Daya S, Dana R, et al. Global consensus on the management of limbal stem cell deficiency. Cornea. 2020;39:1291–302. 32639314 10.1097/ICO.0000000000002358

[3] Deng SX, Borderie V, Chan CC, Dana R, Figueiredo FC, Gomes JAP, et al. Global consensus on definition, classification, diagnosis, and staging of limbal stem cell deficiency. Cornea. 2019;38:364–75. 30614902 10.1097/ICO.0000000000001820 PMC6363877

[4] Vazirani J, Nair D, Shanbhag S, Wurity S, Ranjan A, Sangwan V. Limbal stem cell deficiency-demography and underlying causes. Am J Ophthalmol. 2018;188:99–103. 29378178 10.1016/j.ajo.2018.01.020

[5] Lee YF, Yong DWW, Manotosh R. A review of contact lens-induced limbal stem cell deficiency. Biology. 2023;12:1490. 38132316 10.3390/biology12121490 PMC10740976

[6] Jain R, Sharma N, Basu S, Iyer G, Ueta M, Sotozono C, et al. Stevens-Johnson syndrome: the role of an ophthalmologist. Surv Ophthalmol. 2016;61:369–99. 26829569 10.1016/j.survophthal.2016.01.004

[7] Lee HJ, Colby KA. A review of the clinical and genetic aspects of aniridia. Semin Ophthalmol. 2013;28:306–12. 24138039 10.3109/08820538.2013.825293

[8] Kim KH, Mian SI. Diagnosis of corneal limbal stem cell deficiency. Curr Opin Ophthalmol. 2017;28:355–62. 28426441 10.1097/ICU.0000000000000387

[9] Varma S, Shanbhag SS, Donthineni PR, Mishra DK, Singh V, Basu S. High-resolution optical coherence tomography angiography characteristics of limbal stem cell deficiency. Diagnostics. 2021;11:1130. 34205702 10.3390/diagnostics11061130 PMC8233779

[10] Fukuda R, Usui T, Tomidokoro A, Mishima K, Matagi N, Miyai T, et al. Noninvasive observations of peripheral angle in eyes after penetrating keratoplasty using anterior segment fourier-domain optical coherence tomography. Cornea. 2012;31:259–63. 22189595 10.1097/ICO.0b013e318226daa9

[11] Ono T, Mori Y, Nejima R, Iwasaki T, Miyai T, Aihara M, et al. Comparison of corneal irregularity after recurrent and primary pterygium surgery using Fourier harmonic analysis. Transl Vis Sci Technol. 2021;10:13. 34515760 10.1167/tvst.10.11.13 PMC8444459

[12] Chen LW, Ono T, Hashimoto Y, Tsuneya M, Abe Y, Omoto T, et al. Regular and irregular astigmatism of bullous keratopathy using Fourier harmonic analysis with anterior segment optical coherence tomography. Sci Rep. 2022;12:17865. 36284222 10.1038/s41598-022-22144-w PMC9596404

[13] Tsai TY, Chang HT, Weng SW, Chu CC, Wang YC, Zhao Z, et al. Ocular surface reconstruction of Steven Johnson syndrome / toxic epidermal necrolysis affected eye - a case report. Heliyon. 2023;9:e12590. 36820177 10.1016/j.heliyon.2022.e12590 PMC9938410

[14] Oshika T, Tomidokoro A, Maruo K, Tokunaga T, Miyata N. Quantitative evaluation of irregular astigmatism by Fourier series harmonic analysis of videokeratography data. Invest Ophthalmol Vis Sci. 1998;39:705–9. 9538876

[15] Miyata K, Minami K, Otani A, Tokunaga T, Tokuda S, Amano S. Proposal for a novel severity grading system for pterygia based on corneal topographic data. Cornea. 2017;36:834–40. 28368995 10.1097/ICO.0000000000001193

[16] Minami K, Miyata K, Otani A, Tokunaga T, Tokuda S, Amano S. Detection of increase in corneal irregularity due to pterygium using Fourier series harmonic analyses with multiple diameters. Jpn J Ophthalmol. 2018;62:342–8. 29532273 10.1007/s10384-018-0583-8

[17] Ibrahim OMA, Yagi-Yaguchi Y, Noma H, Tsubota K, Shimazaki J, Yamaguchi T. Corneal higher-order aberrations in Stevens-Johnson syndrome and toxic epidermal necrolysis. Ocul Surf. 2019;17:722–8. 31325631 10.1016/j.jtos.2019.07.006

[18] Chan CC, Holland EJ. Severe limbal stem cell deficiency from contact lens wear: patient clinical features. Am J Ophthalmol. 2013;155:544-549.e2. 23218703 10.1016/j.ajo.2012.09.013

[19] Rossen J, Amram A, Milani B, Park D, Harthan J, Joslin C, et al. Contact lens-induced limbal stem cell deficiency. Ocul Surf. 2016;14:419–34. 27480488 10.1016/j.jtos.2016.06.003 PMC5065783

[20] Schornack MM. Limbal stem cell disease: management with scleral lenses. Clin Exp Optom. 2011;94:592–4. 21517973 10.1111/j.1444-0938.2011.00618.x

[21] Bonnet C, Lee A, Shibayama VP, Tseng CH, Deng SX. Clinical outcomes and complications of fluid-filled scleral lens devices for the management of limbal stem cell deficiency. Cont Lens Anterior Eye. 2023;46:101528. 34728142 10.1016/j.clae.2021.101528 PMC9054946

[22] Oie Y, Sugita S, Yokokura S, Nakazawa T, Tomida D, Satake Y, et al. Clinical trial of autologous cultivated limbal epithelial cell sheet transplantation for patients with limbal stem cell deficiency. Ophthalmology. 2023;130:608–14. 36736434 10.1016/j.ophtha.2023.01.016

